# Chimpanzee mothers, but not fathers, influence offspring vocal–visual communicative behavior

**DOI:** 10.1371/journal.pbio.3003270

**Published:** 2025-08-05

**Authors:** Joseph G. Mine, Laura C. Dees, Claudia Wilke, Erik P. Willems, Zarin P. Machanda, Martin N. Muller, Melissa Emery Thompson, Richard W. Wrangham, Erik J. Scully, Kevin Langergraber, Sabine Stoll, Katie E. Slocombe, Simon W. Townsend

**Affiliations:** 1 Institute for the Interdisciplinary Study of Language Evolution, University of Zurich, Zurich, Switzerland; 2 Department of Evolutionary Anthropology, University of Zurich, Zurich, Switzerland; 3 Department of Animal and Human Ethology, University of Rennes, Rennes, France; 4 Department of Psychology, University of York, York, United Kingdom; 5 Departments of Anthropology and Biology, Tufts University, Medford, Massachusetts, United States of America; 6 Department of Anthropology, University of New Mexico, Albuquerque, New Mexico, United States of America; 7 Department of Human Evolutionary Biology, Harvard University, Cambridge, Massachusetts, United States of America; 8 Institute of Human Origins, Arizona State University, Tempe, Arizona, United States of America; 9 Linguistic Research Infrastructure, University of Zurich, Zurich, Switzerland; 10 Department of Psychology, University of Warwick, Warwick, United Kingdom; New York University, FRANCE

## Abstract

Face-to-face communication in humans typically consists of a combination of vocal utterances and body language. Similarly, our closest living relatives, chimpanzees, produce multiple vocal signals alongside a wide array of manual gestures, body postures and facial expressions. In humans, the ontogenetic development of communicative behavior is known to be heavily influenced by the child’s primary caretakers. In chimpanzees, the extent to which communicative behavior is learned, as opposed to genetically inherited, remains openly debated. Here, we address this issue within the context of multi-modal communication by investigating kinship patterns in the production of visual behaviors alongside vocal signals in wild chimpanzees from the Kanyawara community, Uganda. We report a similarity in the number of visual behaviors combined with vocal signals between individuals who are related via their mother, while no similarity is observed between paternal relatives, in line with the observation that chimpanzee mothers constitute the primary caretakers, while fathers are not involved in parenting. We conclude that the development of this aspect of multi-modal communicative behavior is unlikely to be genetically driven and is rather a result of learning via exposure to social templates, akin to processes involved in the acquisition of human communication.

## Abstract 2

The influence of social versus genetic factors on communication in non-human hominids is not well understood. This study shows that, in line with their central caretaking role, chimpanzees exhibit presumed social influences on communication behavior from their mothers, but not fathers.

## Introduction

A key feature of human communication is that much of it is socially learned [1]. Input from caregivers is crucial for the development of speech [2], and equally so for acquiring the extensive body language that typically accompanies speech [3]. Non-verbal behavior, which includes postures, gaze, gestures, and facial expressions [4], is an integral part of human communication which can enhance, modify, regulate, or even negate the content of linguistic input [5]. Such non-verbal behavior is known to vary cross-culturally, and not just in the quality of bodily movements but also in their number [6]. Thus, in humans, social learning pervades both the acoustic and visual modalities of communication. In line with existing cross-cultural findings in humans, group-specific differences in vocal behavior in other primates including monkeys as well as our closest-living relatives, great apes, have been documented [7–9], implicating a potential role for social learning. However, when comparing communicative behavior across groups, ruling out ecological and genetic confounds remains a key challenge [9–11]. A promising alternative to circumvent these issues is to look for an influence of social partners on communication *within* a group (e.g., maternal relatives [12] or close social affiliates [10]). Taking this approach, we aim to address the lack of evidence that wild apes acquire aspects of their natural communication systems from their caregivers [13], data which are critical to understanding the evolutionary roots of the human capacity to learn aspects of communication socially.

Research into great ape communication has traditionally examined vocal and visual communicative behaviors independently [14,15]. However, this approach is not representative of real communicative events, wherein vocalizations regularly occur alongside other behaviors such as gestures. The importance of a multi-modal method has therefore received an upsurge of interest [16]. Indeed, recent work investigating which vocal and visual behaviors co-occur most frequently during chimpanzee communication has identified over 100 systematic combinations [17], illustrating a hitherto underappreciated flexibility in chimpanzee signal production. How this diverse repertoire of vocal–visual combinations is acquired is unknown.

Within this emerging repertoire of combined vocal and visual behaviors in chimpanzees, a significant, yet currently neglected, role is played by visual components with low salience. These include body postures, gaze direction, changes in body orientation, visible movements, and general actions produced alongside vocalizations. Given the importance of subtle non-verbal behaviors in human communication [4–6], such visual components might also be relevant to chimpanzee receivers. These diverse visual cues, along with established gestural signals and facial expressions, are henceforth referred to collectively as non-vocal behaviors (NVBs). Here, using this NVB framework, we evaluate the role of the social environment in the acquisition of chimpanzee communicative behavior. Specifically, we follow up work in humans by investigating whether the propensity to produce more or fewer vocal–visual combinations during communication events in chimpanzees is socially influenced by primary caregivers [18].

Chimpanzee mothers are the main caretakers of offspring until these are at least 10 years of age [19], and individuals are still heavily exposed to maternal influence and maternal siblings even beyond this time [20]. Thus, matrilineal relatives represent the prime candidates for social learning templates. Previous research has in fact demonstrated that the distribution of one social custom in wild chimpanzees, in this case, high-arm grooming, is best explained by matrilineal relationship, suggesting a role of learning [21]. By contrast, fathers seldom contribute to offspring care and therefore offer fewer social learning opportunities to their kin. Consequently, we predicted that if social learning influences the number of vocal–visual combinations produced during communication, individuals should exhibit more similar levels of vocal–visual production to their mother and matrilineal kin than to their father and patrilineal kin. If, instead, this communicative feature is predominantly under genetic rather than social influence, individuals should be similar to both their maternal and paternal kin [22,23].

## Results

### Maternal and paternal kinship

**Fig 1:**
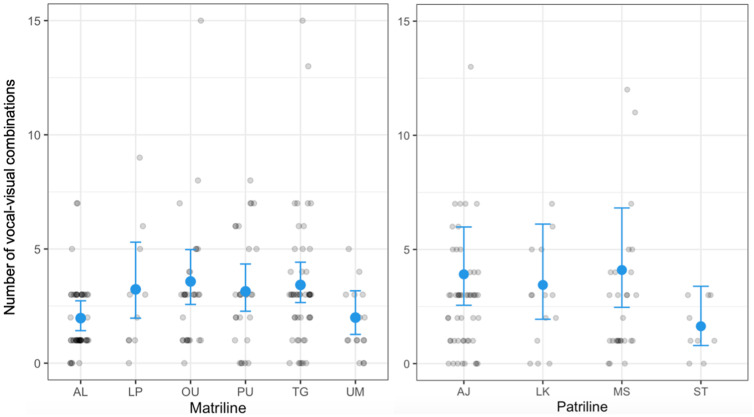
Model predictions showing differences in vocal–visual combination production between maternal and paternal groups. Prediction plots of GLMMs visualizing the variation in number of vocal–visual combinations as a function of kinship groups. Individuals from distinct maternal groups (AL-UM) exhibit different amounts of vocal–visual combinations per event, while individuals from distinct paternal groups (AJ-ST) do not differ. Black dots show raw data, while blue dots show estimated conditional means with associated 95% confidence intervals. Data used for analysis are available in S1 Data.

Visual inspection of the data indicated substantial variation in the number of vocal–visual combinations produced (range 0–15). Furthermore, a GLMM analysis confirmed that maternal kinship was a significant predictor of this variation (*N* events = 182, *N* IDs = 18, *N* matrilines = 6, *χ*^2^_5_ = 15.48, *p* = 0.008; Fig 1 and Table 1). Given the observed amount of individual variation within each matriline, we did not expect robust differences between every matriline and indeed post-hoc pairwise comparisons confirmed this (see S1 Text). In contrast to maternal kinship, paternal kinship had no significant effect (*N* events = 103, *N* IDs = 13, *N* patrilines = 4; *χ*^2^_3_ = 5.01, *p* = 0.171; Fig 1 and Table 1).

**Table 1:** Analysis of deviance tables showing results of likelihood ratio tests for matrilineal and patrilineal models. *χ*^2^ and *p-*values are displayed for all fixed effects (and their interactions) included in the GLMM, as well as for the overall model, including sample sizes. Effect sizes (partial *R*-squared values) are displayed for all fixed effects (and any interactions) included in the GLMM.

Matriline model					Patriline model				
	*χ* ^2^	Df	*p*-value	Partial *R*^2^		*χ* ^2^	Df	*p*-value	Partial *R*^2^
Matriline	15.48	5	0.008	0.08	Patriline	5.01	3	0.171	0.04
Call	9.41	6	0.151	NA	Call	32.85	6	*p* < 0.001	0.02
Duration	5.23	1	0.022	NA	Duration	1.85	1	0.172	0.35
Call:duration	14.01	6	0.029	0.16					
*N* = 182, ID *N* = 18, overall *χ*^2^ = 42.89,					*N* = 103, ID *N* = 13, overall *χ*^2^ = 35.91,				
*p* = 0.0008, pseudo *R*^2^Δ = 0.26					*p* < 0.001, pseudo *R*^2^Δ = 0.39				

### Call type and duration

We controlled for the effect of call type and call duration, which also influenced the number of vocal–visual combinations per event. In the matriline model, a significant interaction was observed between call type and duration (*χ*^2^_6_ = 14.01, *p* = 0.029), such that the relationship between call duration and number of vocal–visual combinations was positive in some call types (e.g., soft hoo, pant hoot) but negative in others (e.g., scream, pant bark; see Fig A in S1 Text for further details). In the patriline analysis, this interaction term was not significant, and only variation in call type was shown to influence the number of vocal–visual combinations (*χ*^2^_6_ = 32.85, *p* < 0.001).

## Discussion

Our results suggest that variation in the number of vocal–visual combinations produced per communicative event in chimpanzees is predicted by maternal kinship, with individuals from the same matriline producing similar numbers of vocal–visual combinations to each other. In contrast, variation in the number of vocal–visual combinations produced was not explained by paternal kinship. Given that chimpanzee infants are raised exclusively by their mothers, our findings suggest that mothers, but not fathers, offer their kin a social template from which communicative behavior can be learned [22]. These findings therefore suggest a potential role of social learning in the development of multi-modal communicative behavior in chimpanzees. Intriguingly, as all chimpanzees observed in this study were aged 10 or older, this corroborates the notion that maternal influences on behavioral development persist into an advanced age, as found for high-arm grooming styles [21].

Despite these results, an alternative explanation might invoke genetic inheritance of communication-related traits through the female X chromosome or mitochondrial DNA (mtDNA). However, such a genetically encoded behavioral profile would be implausible. First, mtDNA genes are generally associated with basic cell metabolism, not complex communicative behavior [24]. Moreover, inheritance via X chromosomes would predict differential expression of vocal–visual tendencies in males and females [25]. Specifically, males would be more likely than females to exhibit similarity to their mother, given that they possess only one copy of the X chromosome. However, such a sex-biased similarity is not present in our sample (see S1 Text for further details).

It is important to note that the scope of the current study is limited to a single dimension of chimpanzee communication, namely the number of vocal–visual combinations produced per event. What function this variation in number of combinations per event might have currently remains speculative. For instance, by using a greater number of vocal–visual combinations, individuals may incrementally refine their signals either to increase redundancy or to achieve greater nuance [26]. However, what has not been addressed here is whether the type of multi-modal signals, specifically which vocal and visual behaviors are combined, also differs as a function of maternal versus paternal kinship. The current dataset was insufficient to perform such analyses, which would have required more instances of the many different signal types for reliable assessment. Thus, a detailed analysis of the acquisition of specific vocal–visual combinations in addition to disentangling the function of variation in number of NVBs accompanying vocalisations, remain important objectives for future studies addressing the role of social learning in great ape communication.

Previous work has offered similar evidence for the social learning of communication in other primate species [8,9,27]. However, ruling out more parsimonious explanations driving variation in communicative behavior, such as genetic similarity or shared ecology, has remained a challenge. A well-established approach to decomposing phenotypic variation into genetic and environmental sources is a quantitative genetics method known as the ‘animal model’ [28]. However, this approach could not be feasibly implemented here as it requires well-connected pedigrees of hundreds of individuals, which are challenging to obtain in wild apes even from long-term databases [29]. Our study highlights a promising alternative paradigm for disentangling the socially learned and genetic underpinnings of chimpanzee behavior by measuring similarity to maternal and paternal kin, using data more typical of wild settings.

A key implication of our findings is therefore that a hallmark of human communication, namely the social acquisition of certain aspects of communicative behavior (in this case the tendency to produce more versus fewer vocal–visual combinations), might be phylogenetically more ancient than previously assumed. Future efforts to replicate these findings across other great apes, particularly bonobos, our other closest-living relative, are central to confirming this hypothesis and ruling out convergent evolutionary processes.

## Methods

### Study site and data collection

The study was conducted on wild chimpanzees from the Kanyawara community in Kibale National Park, Uganda [30]. The population includes ~60 individuals inhabiting a home range of ~15 km^2^. The Kanyawara community has been the object of long-term study since 1987 and is entirely habituated. The data used here were collected between February and May 2013, and between June 2014 and March 2015 [31]. These data consist of video-audio recordings made within the chimpanzee home range, between 0800 and 1900 hours. The equipment used was a hand-held video recorder (Panasonic HDC-SD90), connected to an external microphone (Sennheiser MKE 400).

Individuals were recorded from a distance of at least 7 m while engaged in their natural behavior. We used focal animal sampling, involving 15 min of continuous video observation of one animal, intended to capture a clear and complete view of the animal and all its behaviors, including communication. Focal animals were only sampled once per day.

### Behavioral annotation and inter-observer reliability

Using Observer XT 10 video coding software (http://www.noldus.com/animal-behaviour-research), we annotated observational video/audio footage of 210 communication events from 12 males and 10 females, between the ages of 10 and 48. We extracted information on maternal and paternal kinship of individuals from the long-term database of the Kanyawara community [30]. Maternal kinship data were available for 18 out of 22 individuals, for a total of 6 matriline groups and 182 events. Paternal kinship data were available for 13 out of 22 individuals, for a total of 4 patriline groups and 103 events.

As outlined in Mine and colleagues 2024 [17], vocalizations were categorized in line with published chimpanzee vocal repertoires and empirical work [32,33]. Of the ~13 established call types, we focused on the seven types that occurred most frequently: grunt, soft hoo, pant bark, pant grunt, pant hoot, scream, and whimper. To be included in the analysis, a call type needed to occur a minimum of five times. Additional call types that were not observed at least five times and therefore excluded from the study were the following: bark, waa bark, pant, cough, wraa, laughter, squeak. Chimpanzee vocalizations frequently occur in bouts. We defined a bout as a repeated emission of the same call type with pauses shorter than 10 s between the individual units. A bout was considered ended when followed by a silent interval of 10 s or by the production of a different call type. Bouts constituted single data points. The duration of vocal bouts ranged between 1–62 s.

In association with each vocal bout, NVBs were recorded. NVBs were only annotated while a vocalization bout was ongoing. We recorded a total of 31 different NVB types. Table 2 adapted from [17] provides the full list of NVBs annotated in this study, as well as a description of the behavioral criteria used to classify NVBs. We then quantified the number of vocal–visual combinations for each of the 210 vocalization events. To exclude chance combinations of vocal signals and NVBs, only combinations which were shown to occur at above chance level via collocation analysis [17,34] were included in the analysis. Up to 15 of these significant vocal–visual combinations were recorded for each event.

**Table 2:** Full list of NVBs annotated in Mine and colleagues 2024 [17] and in this study with corresponding behavioral description used to assign NVBs. The term “specific individual” used above refers to the individual who is closest to the signaler.

NVB name	NVB description
rest	Signaler is in lying down or resting position with chest or back touching the ground
sit	Signaler sits with bottom touching ground, chest or back not touching ground
get_up	Signaler transitions from lying or sitting position to standing or walking
stand	Signaler is in erect quadrupedal position without movement
walk	Signaler moves quadrupedally by more than 1 m
run	Quadrupedal movement that occurs at a faster pace than normal walking, often gallop-like appearance with both feet in the air at once
climb	Signaler moves up, down or along the trunk or branch of a tree
look_towards	Head orientation is shifted toward specific individual by at least 90° resulting in specific individual being in line of sight of signaler
look_away	Head orientation shifted away from specific individual by at least 90°
gaze_upwards	Head orientation is shifted towards the canopy/sky
gaze_alternation	Head orientation changes three or more times by approximately 90°
turn_body_towards	Body orientation changed by at least 90° in direction of specific individual
turn_body_away	Body orientation is shifted away from specific individual by at least 90°
extend_body_towards	Signaler moves chest, back or bottom toward a specific individual but legs do not usually move
retract_body	Signaler’s body axis connecting hips to head either changes angle or moves away from specific individual
crouch_down	Signaler brings bottom, body or shoulders close to the ground
present_back	Signaler orients back and bottom toward a specific individual by at least 90°
arm_reach	Arm is fully or partially extended towards a specific individual with or without contact
arm_wave	Arm performs repetitive back and forth or side to side motion
scratch_self	Fingers perform loud scratching gesture against any body surface
approach	Signaler moves in direction of specific individual with 45° accuracy on either side
embrace	Arms or legs are wrapped around a specific individual with degree of surface body contact being at least hand/foot + forelimb
chase	Signaler runs or climbs quickly after a specific individual in aggressive manner
hit	Hand or foot is moved aggressively with the intent to make contact with body part of another individual
grab branch	Tree branch is grabbed and shaken or dragged along the floor while running or displaying
slap_ground	Hands or feet are brought violently against the ground to produce a smacking noise, sometimes repeatedly
feed	Signaler grabs food items and places in mouth, or chews food items already in mouth
groom	Signaler probes own hair or that of other individual and extracting small particles, using one or both hands
play	Signaler interacts with another individual via non-aggressive grabbing, biting, chasing, climbing, tickling
relaxed_open_mouth_face	Open mouth with intermediate separation between upper and lower jaw, while engaged in play
scream_face	Wide open mouth with maximum separation between upper and lower jaw, lip corners pulled up, teeth bared

To ensure videos were coded reliably, a second observer coded 11% of the events and annotated the call type (at least one call for each call type was present in the subset) as well as NVBs (at least one instance of each NVB type was coded in the subset). A Cohen’s kappa value of 0.82 and 0.88 for vocalization type and NVB type, respectively, was computed, indicating excellent levels of agreement in both cases [35].

### Statistical analyses

We implemented Generalized Linear Mixed Models with a negative binomial error structure and log link function. We included matriline or patriline, along with call duration and call type as predictors, individual identity nested within matriline/patriline as random factors, and the number of significant NVB-vocalization combinations for each event as response. With this model structure, the effect of any predictor on the response controls for the potentially confounding influence of the other predictors. It is worth noting that some call types were sparsely represented in the dataset, and thus exhibited higher uncertainty around parameter estimates. Demographic variables such as age, sex and rank were previously shown to have no effect on the number of NVBs alongside vocalizations [17], and therefore were excluded from the statistical model. Model assumptions, checked using the DHARMa package in R, were met.

### Ethical statement

This study complied with the ASAB/ABS guidelines for the use of animals in research; ethical approval was granted by the Biology Ethics Committee (University of York). The Biology Ethics Committee at the University of York issues letters of approval (see S1 Ethics), but not approval numbers. As this work is purely observational, no home office/UK permit/protocol/project license was required. The Ugandan Wildlife Authority and the Ugandan National Council for Science and Technology granted consent to carry out the data collection in Uganda. Non-invasive observational video/audio data were recorded from a minimum distance of 8 m from the chimpanzee subjects, to minimize the risk of human disease transmission and to avoid interference with the subjects’ natural behavior.

## Supporting information

S1 Text Post-hoc comparisons of matriline groups; Ruling out of low-level explanations for matrilines showing significant differences; Fig A in S1 Text (illustrating interaction term between call type and duration); Test of interaction between matriline and sex; References for Supporting information.
(DOCX)

S1 Data Datasets for analyses performed in this study. (CSV)

S1 File Legend for S1 Data. (CSV)

S1 Code R code for analyses performed in this study. (DOCX)

S1 Ethics Ethics approval letter. (PDF)

## Abbreviations

mtDNA: mitochondrial DNA
NVBs: non-vocal behaviors
