# Supplementary material for: Chimpanzee mothers, but not fathers, influence offspring vocal–visual communicative behavior
Source: PLoS Biol. 2025 Aug 5;23(8):e3003270. doi: 10.1371/journal.pbio.3003270 (PMC12324129; doi:10.1371/journal.pbio.3003270)
Supplement: S1 Text — Post-hoc comparisons of matriline groups; Ruling out of low-level explanations for matrilines showing significant differences; Fig A in S1 Text (illustrating interaction term between call type and duration); Test of interaction between matriline and sex; References for Supporting information. (DOCX) [file pbio.3003270.s001.docx]

S1 Text: Supporting Information for

“Chimpanzee mothers, but not fathers, influence offspring vocal-visual communicative behaviour”

**This S1 Text file includes:**

- Post-hoc comparisons of matriline groups
- Ruling out of low-level explanations for matrilines showing significant differences
- Fig A in S1 Text: further information on interaction term between call type and duration
- Test of interaction between matriline and sex
- References for Supporting Information

**Other Supporting Information files for this manuscript include the following:**

- S1 Data: datasets for analyses performed in this study
- Legend for S1 Data
- S1 Code: R code for analyses performed in this study
- Ethics approval letter

**Post-hoc comparisons of matriline groups**

Post-hoc pairwise comparisons revealed that matriline AL differs significantly from matrilines OU and TG (estimate = -0.59, SE = 0.19, p = 0.024 and estimate = -0.55, SE = 0.17, p = 0.016, respectively). As predicted, given individual variation and the fact that the response variable is continuous, we did not detect substantial differences between each matriline but rather subtle difference between most matrilines with significant variation at the extremes of the continuum.

To explore the likelihood that significant differences between matrilines (AL, OU and TG) resulted from lower-level confounding effects related to, for example, the sampling of behavioural contexts for these matrilines or their demographic characteristics (e.g. rank, age), we also performed a number of follow-up exploratory analyses.

First, the sampling effort for the following matrilines was similar:

- Matriline AL: 50 events
- Matriline OU: 30 events
- Matriline TG: 48 events

Secondly, as chimpanzees are known to modify their communicative behaviour depending on the composition of the audience [1,2], we examined the party size, i.e. the available audience for signal reception, in events from matrilines AL, OU and TG:

- Matriline AL: party size ranges between 2-7, mean 3.1
- Matriline OU: party size ranges between 2-8, mean 3.2
- Matriline TG: party size ranges between 2-6, mean 3.2

Next, we probed whether the behavioural context of signal production, defined as the interactions occurring between signaler and receiver in the 60 seconds prior to vocalization, varied across events recorded for matrilines AL, OU and TG. We report the percentage of communicative events which occurred in each behavioural context:

- Matriline AL: feeding 20%, grooming 28%, travel 40%, resting 6%, other 6%
- Matriline OU: feeding 20%, grooming 30%, travel 20%, resting 17%, other 13%
- Matriline TG: feeding 31%, grooming 13%, travel 21%, resting 21%, other 14%

Finally, we examined the homogeneity between matrilines in terms of call type production, rank and age. It is worth noting that the effect of these variables on NVB production was already explicitly addressed via inclusion in statistical models, in the current study as well as in Mine et al. 2024 [3] nonetheless, below we report these variables:

Call type production:

- Matriline AL: grunt 20%, pant grunt 16%, pant hoot 6%, soft hoo 40%, 6 out of 7 call types represented
- Matriline OU: grunt 30%, pant grunt 4%, pant hoot 23%, soft hoo 30%, 6 out of 7 call types represented
- Matriline TG: grunt 40%, pant grunt 6%, pant hoot 12%, soft hoo 23%, 6 out of 7 call types represented

Rank

- Matriline AL: average rank 9.6
- Matriline TG: average rank 9.8
- Matriline OU: average rank 13.6
- All other matrilines: average 9.01

Age

- AL: average age 18 years
- OU: average age 26 years
- TG: average age 16 years
- All other matrilines: average age 24 years

Thus overall, the matrilines which varied the most do not appear to differ dramatically in any of the ways explored.

**Interaction term between call type and duration**


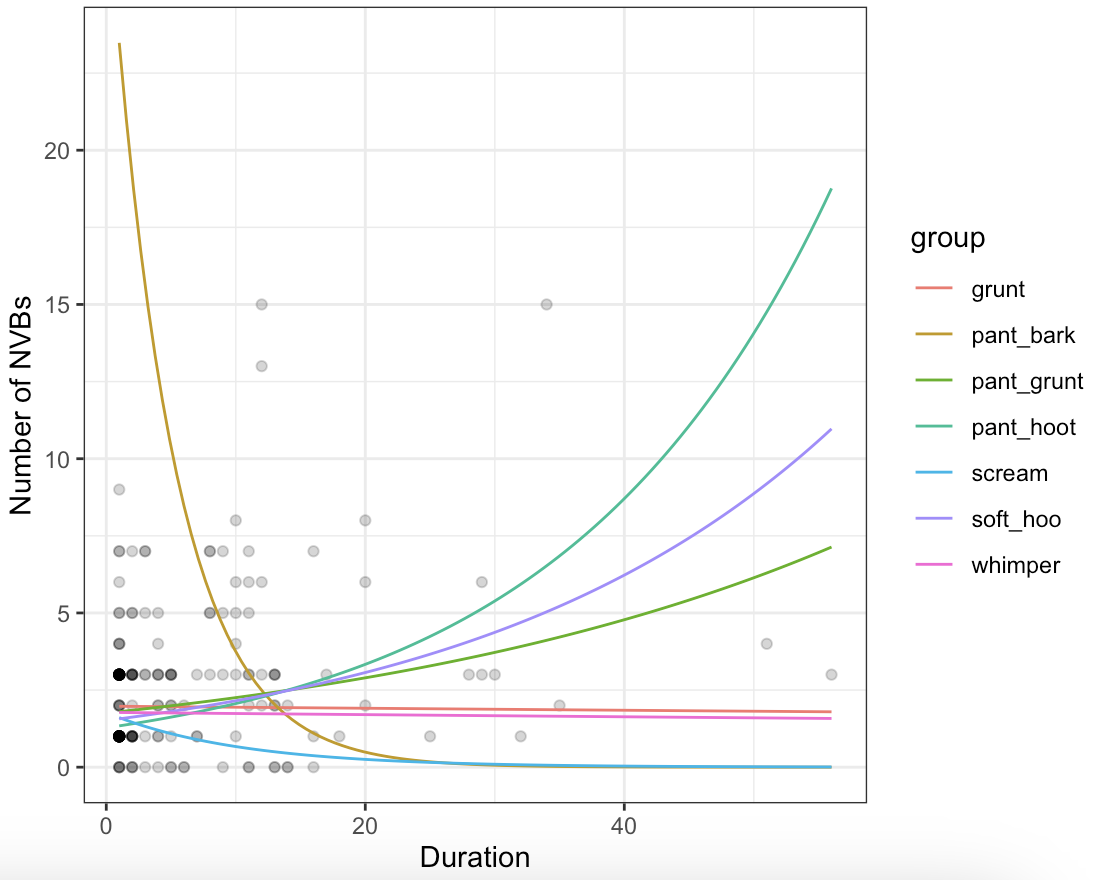


**Fig A in S1 Text.** In the matriline model, a significant interaction was observed between the predictors “duration” and “call type” on the number of NVBs produced per vocalization event. The figure illustrates the differential effect of duration on the number of NVBs depending on call type. Crucially, all 3 of the matrilines which were found to differ significantly in terms of NVB number (matrilines AL, OU and TG) included multiple call types that were positively as well as negatively associated with NVB number. This further supports the notion that the observed differences between matrilines is not merely driven by low-level explanations such as differences in call duration between matrilines.

**Interaction term between matriline and sex**

As outlined in the discussion, an additional alternative explanation of our results posits that the observed matrilineal differences may be explained by inheritance via sex chromosomes. Such a mechanism of inheritance would predict differential expression of the trait in males and females. In order to explicitly address this alternative explanation, we fitted a model which includes an interaction term between the predictors “sex” and “matriline”. To generate this model, it was necessary to exclude individuals from the matriline PU from the sample, as this matriline contained only male individuals and would therefore impede the interaction term between matriline and sex. In the output of this model, the main effect of matriline is retained (χ^2^_4_ = 14.04, p = 0.007), yet there is no significant interaction between matriline and sex (χ^2^_4_ = 3.2, p = 0.511), nor any significant first-order effect of sex (χ^2^_1_ = 0.49, p = 0.481). Thus, differential expression in males and females of the behaviour in question, i.e. the production of non-vocal behaviours alongside vocalizations, is not supported by our data.

**References**

1. Townsend, S. W., & Zuberbuhler, K. (2009). Audience effects in chimpanzee copulation calls. *Communicative & integrative biology*, *2*(3), 282-284.
2. Slocombe, K. E., & Zuberbühler, K. (2007). Chimpanzees modify recruitment screams as a function of audience composition. *Proceedings of the National Academy of Sciences*, *104*(43), 17228-17233.
3. Mine, J. G., Wilke, C., Zulberti, C., Behjati, M., Bosshard, A. B., Stoll, S., ... & Townsend, S. W. (2024). Vocal-visual combinations in wild chimpanzees. *Behavioral Ecology and Sociobiology*, *78*(10), 1-13.
