# Supplementary material for: Chimpanzee mothers, but not fathers, influence offspring vocal–visual communicative behavior
Source: PLoS Biol. 2025 Aug 5;23(8):e3003270. doi: 10.1371/journal.pbio.3003270 (PMC12324129; doi:10.1371/journal.pbio.3003270)
Supplement: S1 Code — (DOCX) [file pbio.3003270.s004.docx]

R code for Mine et al. submission to PLOS Biology

## Prepare session ----------------------------------------------------------

library(ggpubr)

theme_set(theme_bw())

library(glmmTMB)

library(car)

library(DHARMa)

library(MuMIn)

library(emmeans)

library(ggeffects)

## Import data -----------------------------------------------------------------

dat3<- read.csv("DATA_Mine_et_al.csv", stringsAsFactors= T)

## Fit matriline model of interest -------------------------------------------------------

mod3.00<- glmmTMB(collocates~ 1 + (1|matriline/ID),

data= dat3, family= nbinom2(link= "log"))

mod3<- glmmTMB(collocates~ matriline + call * duration + (1|matriline/ID),

data= dat3, family= nbinom2(link= "log"))

mod33<- glmmTMB(collocates~ call * duration + (1|matriline/ID),

data= dat3, family= nbinom2(link= "log"))

mod333<- glmmTMB(collocates~ matriline + (1|matriline/ID),

data= dat3, family= nbinom2(link= "log"))

anova(mod3.00, mod3)

summary(mod3)

Anova(mod3); r.squaredGLMM(mod3)

Anova(mod33); r.squaredGLMM(mod33)

Anova(mod333); r.squaredGLMM(mod333)

anova(mod33, mod3)

emmeans(mod3, pairwise~ matriline)

## Fit patriline model of interest -------------------------------------------------------

modp3.00<- glmmTMB(collocates~ 1 + (1|patriline/ID),

data= dat3, family= nbinom2(link= "log"))

modp3<- glmmTMB(collocates~ patriline + call + duration + (1|patriline/ID),

data= dat3, family= nbinom2(link= "log"))

modp33<- glmmTMB(collocates~ call + duration + (1|patriline/ID),

data= dat3, family= nbinom2(link= "log"))

modp333<- glmmTMB(collocates~ patriline + call + (1|patriline/ID),

data= dat3, family= nbinom2(link= "log"))

modp3333<- glmmTMB(collocates~ patriline + duration + (1|patriline/ID),

data= dat3, family= nbinom2(link= "log"))

anova(modp3.00, modp3)

summary(modp3)

Anova(modp3); r.squaredGLMM(modp3)

Anova(modp33); r.squaredGLMM(modp33)

Anova(modp333); r.squaredGLMM(modp333)

Anova(modp3333); r.squaredGLMM(modp3333)

emmeans(modp3, pairwise~ patriline)

# DHARMa

sim.res<- simulateResiduals(mod3, n= 1e3)

plot(sim.res)

hist(sim.res)

sim.res<- simulateResiduals(modp3, n= 1e3)

plot(sim.res)

hist(sim.res)

## matriline prediction plots -----------------------------------------

# Create a vector to create nice smooth lines

d3<- seq(min(mod3$frame$duration), max(mod3$frame$duration), length.out= 1e2)

pred.mat<- ggpredict(mod3, "matriline")

pred.dur<- ggpredict(mod3, c("duration [d3]", "call"))

ggarrange(

ggplot(pred.mat, aes(x, predicted)) +

geom_jitter(data=mod3$frame, aes(matriline, collocates), height=0, width= .2,

alpha= .2) +

geom_errorbar(aes(ymin= conf.low, ymax= conf.high), width= .2, col= 4) +

geom_point(size= 3, col= 4) +

labs(x="Matriline", y= "Number of NVBs"),

ggplot(pred.dur, aes(x, predicted)) +

geom_point(data=mod3$frame, aes(duration, collocates), alpha= .2) +

# geom_ribbon(aes(ymin= conf.low, ymax= conf.high, fill= group), alpha= .2) +

geom_line(aes(col= group)) +

labs(x= "Duration", y= "Number of NVBs", group= "Call type"),

ncol= 2)

## patriline prediction plots -----------------------------------------------------

# Create a vector to create nice smooth lines

dp3<- seq(min(modp3$frame$duration), max(modp3$frame$duration), length.out= 1e2)

pred.matp<- ggpredict(modp3, "patriline")

pred.durp<- ggpredict(modp3, c("duration [dp3]", "call"))

ggarrange(

ggplot(pred.matp, aes(x, predicted)) +

geom_jitter(data=modp3$frame, aes(patriline, collocates), height=0, width= .2,

alpha= .2) +

geom_errorbar(aes(ymin= conf.low, ymax= conf.high), width= .2, col= 4) +

geom_point(size= 3, col= 4) +

labs(x="Patriline", y= "NVB") +

coord_cartesian(ylim= c(0, 15)),

ggplot(pred.dur, aes(x, predicted)) +

geom_point(data=modp3$frame, aes(duration, collocates), alpha= .2) +

# geom_ribbon(aes(ymin= conf.low, ymax= conf.high, fill= group), alpha= .2) +

geom_line(aes(col= group)) +

labs(x= "Duration", y= "NVB", group= "Call type")+

coord_cartesian(ylim= c(0, 15)),

ncol= 2)
