# Supplementary material for: Chimpanzee mothers, but not fathers, influence offspring vocal–visual communicative behavior
Source: PLoS Biol. 2025 Aug 5;23(8):e3003270. doi: 10.1371/journal.pbio.3003270 (PMC12324129; doi:10.1371/journal.pbio.3003270)
Supplement: S1 Ethics — (PDF) [file pbio.3003270.s005.pdf]

THE UNIVERSITY of York

Memorandum

To: Dr Katie Slocombe

From: Pat Coulson; Secretary Biology Ethics Committee

Date: 27/11/12

Re: Ethical Approval

Dear Katie,

Re project entitled: Do more cooperative chimpanzees (*Pan troglodytes*) show more complex multimodal communication?

Thank you for submitting the above project for consideration by the Department of Biology Ethics Committee.

The information you provided has been evaluated and I am pleased to approve the application on behalf of the Committee.

Yours sincerely

Professor Paul Kaye (Chair)

Department of Biology Ethics Committee
University of York
